# IL-1 polymorphisms modulates functional recovery following rehabilitation in multiple sclerosis

**DOI:** 10.3389/fimmu.2026.1872267

**Published:** 2026-06-30

**Authors:** Cristina Agliardi, Franca Rosa Guerini, Milena Zanzottera, Elisabetta Bolognesi, Domenico Caputo, Elisabetta Groppo, Marco Rovaris, Mario Clerici

**Affiliations:** 1 Laboratory of Molecular Medicine and Biotechnology (LAMMB), IRCCS Fondazione Don Carlo Gnocchi, Milan, Italy; 2 Neurology Unit, IRCCS Fondazione Don Carlo Gnocchi, Milan, Italy; 3 Neurology Unit, GOM Niguarda, Milan, Italy; 4 Pathophysiology and Transplantation Department, University of Milan, Milan, Italy

**Keywords:** EDSS, genetics, IL-1 polymorphisms, multiple sclerosis, rehabilitation, SNPs

## Abstract

**Background:**

Interleukin-1 (IL-1) cytokines as well as IL-1 genes are key regulators of neuroinflammation in Multiple Sclerosis (MS), with emerging roles in synaptic plasticity and tissue repair. While genetic determinants of MS susceptibility have been extensively characterized, their contribution to neurofunctional recovery remains poorly understood.

**Objective:**

To investigate whether functional polymorphisms in IL-1 genes influence rehabilitation outcomes in MS, providing insight into immunogenetic determinants of recovery.

**Methods:**

We analyzed by Allelic Real-time PCR IL-1α rs3783521 and IL-1β rs16944, rs1143627, and rs1143634 polymorphisms in 162 MS patients undergoing inpatient multidisciplinary rehabilitation without concomitant disease-modifying therapies. Disability was assessed using the Expanded Disability Status Scale (EDSS) and Modified Barthel Index (BI) at admission and discharge. Changes in disability (ΔEDSS, ΔBI) were evaluated using non-parametric tests and multivariable generalized linear models adjusted for clinical covariates.

**Results:**

IL-1β rs1143634 was associated with MS susceptibility, with the A allele conferring increased risk. All polymorphisms significantly influenced ΔEDSS in univariate analyses. In adjusted models, IL-1α rs3783521 and IL-1β rs1143634 A alleles were independently associated with greater functional improvement, whereas IL-1β rs16944 A allele was associated with reduced recovery. IL-1β rs1143627 showed a significant effect under a dominant model. Notably, combined carriage of IL-1α rs3783521 A and IL-1β rs16944 G alleles identified a subgroup with the greatest rehabilitation response. No consistent associations were observed for ΔBI.

**Conclusion:**

IL-1α rs3783521, IL-1β rs16944 polymorphisms contribute to inter-individual differences in neurofunctional recovery following rehabilitation in MS. These findings support a role for neuroinflammatory genetic profiling in predicting rehabilitation outcomes and highlight immune-mediated mechanisms as modulators of CNS repair. This work provides a translational framework for integrating immunogenetics into personalized rehabilitation strategies.

## 1 Introduction

Multiple sclerosis (MS) is a neuroinflammatory disease of the central nervous systems (CNS) that predominantly affects young adults, mainly females, with an overall ratio of 3:1 (females:males). MS is characterized by demyelination and neurodegeneration with consequent disability and substantial decrease in quality of life (1). MS presents with different clinical courses; the most frequent phenotype is relapsing remitting-MS (RR-MS) (85-90% of total new diagnoses), characterized by acute neurological relapses (neurological symptoms and signs lasting at least 24 hours) alternated by remissions, that is periods of clinical stability. Fifteen to thirty percent of RR-MS patients evolve in another clinical phenotype during disease course: secondary progressive-MS (SP-MS), characterized by disability worsening without evidence of new acute inflammation both at neurological assessment and at MRI. Finally, 10-15% of patients present with a primary progressive-MS (PP-MS) phenotype at disease onset, characterized by slow and constant disability progression (2). MS pathogenesis includes a complex interplay of genetic susceptibility and environmental factors (3). Monozygotic twins have a 20-30% concordance rate compared to dizygotic twins (2-5%) providing a strong support for a genetic aetiology in MS (4–6). Genome-wide association studies (GWAS) and targeted genomic screens suggested a polygenic model of MS heritability with more than 200 independent regions across the genome being associated with susceptibility (7). Nevertheless, the strongest genome-wide disease association signal maps to the 4-Mb major histocompatibility complex (MHC) region on chromosome 6p21.1, with HLA-DRB1*15:01 representing the major susceptibility allele for MS.In addition, several non-MHC genes involved in immune regulation, including IL2RA and IL7R, have been associated with MS susceptibility suggesting a role in the modulation of inflammatory signaling as well of as immune responses within the CNS (8, 9). Genetics not only influences MS susceptibility but also many other aspects of the pathology such as the development of clinical phenotypes, disease activity and progression (10) as well as response to disease modifying pharmacological (11) and rehabilitative treatments (12, 13). Several biomarkers are currently used to support MS diagnosis, monitor disease activity, and assess progression. MRI remains the gold standard for detecting inflammatory lesions and evaluating disease dissemination in space and time (1). In addition, cerebrospinal fluid (CSF) oligoclonal bands (OCBs) represent a well-established diagnostic biomarker reflecting intrathecal immunoglobulin synthesis, while the measurement of neurofilament light chain (NfL) in the CSF has emerged as a promising marker of neuroaxonal damage and disease activity. Glial fibrillary acidic protein (GFAP) has been proposed as a marker of astrogliosis as well (14). However, despite their clinical utility, these biomarkers only partially capture the complex immunopathological mechanisms underlying MS heterogeneity, progression, and recovery potential. In particular, currently available biomarkers are limited in their ability to predict individual functional outcomes and response to rehabilitative interventions. In this context, increasing attention has been directed toward immunological and genetic biomarkers involved in neuroinflammation and tissue repair. Among these, the interleukin (IL-1) cytokine family is of particular interest because of its dual role in inflammatory responses and modulation of synaptic plasticity (15).

From an immunological perspective, MS is driven by autoreactive T helper (Th) cells that are responsible for demyelination and axonal damage. Moreover, aberrant stimulation of B cells and plasma cells could also inflict damage by producing autoantibodies against specific myelin antigens (16). Several members of the IL-1 family of cytokines have been studied in the context of experimental autoimmune encephalomyelitis (EAE), the murine model of MS, and as well as in MS itself. MS patients, in fact, exhibit elevated IL-1β and/or an increased IL-1β/IL-1ra ratio in the cerebrospinal fluid (CSF) and in peripheral blood (17). IL-1β, whose expression is tightly regulated, has been detected in white matter and within acute lesions (18), and its presence is associated with the extent of cortical lesion burden (19). Moreover, both microglia- and blood-derived macrophages that infiltrate the CNS in MS patients produce high amounts of IL-1β (20). IL-1β and IL-1α, which is constitutively expressed, bind to the same receptor, the IL-1 type 1 receptor (IL-1R1), and have partially overlapping inflammatory activities activating both innate and adaptive immune pathways (21, 22). We hypothesize that IL-1α and IL-1β genetic polymorphisms may influence interindividual variability in functional outcomes following rehabilitation in MS patients. To test this hypothesis, we analyzed *IL-1α rs3783521*, *IL-1β rs16944, rs1143627* and *rs1143634* single nucleotide polymorphisms (SNPs) in a group of MS patients undergoing inpatient multidisciplinary rehabilitation who were selected for not taking any pharmacological treatment during the rehabilitation period to avoid potential drug-related confounding effects.

## 2 Materials and methods

### 2.1 Study population

A total of one-hundred-sixty-two subjects with a diagnosis of MS (30 PP-MS, 43 RR-MS and 89 SP-MS) according to the revised McDonald diagnostic criteria were enrolled in this study (23). The MS patients who were eligible for the present study were selected from a larger cohort of a previous study (24) who underwent an inpatient multidisciplinary rehabilitation treatment at Neurorehabilitation Unit, MS Centre, IRCCS Fondazione Don C. Gnocchi (Milan, Italy). Because IL-1 polymorphisms are associated with several inflammatory and autoimmune conditions, patients with relevant comorbid autoimmune or chronic inflammatory diseases were excluded to minimize potential confounding effects. Moreover, only patients with MS who were not receiving pharmacological treatment during the study period were included, thereby reducing the potential influence of treatment-related factors on rehabilitation outcomes.

Admission criteria to the rehabilitation program and the rehabilitation regimen are described in Groppo E et al. To evaluate the outcome of the rehabilitation procedures, the Modified Barthel Index (mBI) (25) and the Expanded Disability Status Scale (EDSS) (26) were assessed both at admission and at discharge (from now named as BI A, BI D, EDSS A, EDSS D). mBI is a widely used measure of functional independence in activities of daily living, assessing domains such as mobility, self-care, and continence, scores range from 0 to 100, with higher values indicating greater functional independence. EDSS is the most commonly used clinical scale for quantifying disability in multiple sclerosis. It ranges from 0 (normal neurological examination) to 10 (death due to MS), with higher scores reflecting greater disability and greater impairment in ambulation and neurological function. The study was conducted in accordance with the principles of the Declaration of Helsinki and was approved by the institutional review board (protocol number #11_27/06/2019). All participants gave written informed consent.

### 2.2 Sample collection and DNA extraction

Whole blood was collected by venepuncture in EDTA-containing Vacutainer tubes (Becton Dickinson Co., USA) from all study participant. Genomic DNA was extracted by phenol/chloroform method. DNA concentration and purity were measured by NanoVue spectrophotometer (GE HealthCare, USA). DNA was then stored at -20 °C until use.

### 2.3 IL-1α rs3783521, IL-1β rs16944, rs1143627 and rs1143634 SNPs description and genotyping

*IL-1α rs3783521*, *IL-1β rs16944*, *rs1143627* and *rs1143634* SNPs were selected based on their functional relevance and prior evidence demonstrating significant associations with autoimmune diseases (27–29). *IL-1α rs3783521 G>A*, *IL-1β rs16944 A>G* and *rs1143627 A>G* are located in the promoter region of the gene, *IL-1β rs1143634 G>A* instead is in exon 5. All the SNPs were analyzed by allelic discrimination Real-time PCR by the use of the following TaqMan probes (Thermofisher scientific, USA): C:_1839900_10 for *rs3783521* (A=VIC; G=FAM), C:_1839943_10 for *rs16944* (G=VIC; A=FAM), C:_1839944_10 for *rs1143627* (G=VIC; A=FAM) and C:_9546517_10 for *rs1143634* (G=VIC; A=FAM). The PCR protocol consisted of an initial hot start at 95 °C for 10 minutes, followed by 40 cycles of 15 seconds at 94 °C and 1 minute at 60 °C. Fluorescence detection was performed at 60 °C. Reactions were carried out in 10 μl volumes using TaqMan Genotyping Master Mix (Thermo Fisher Scientific) on 96-well plates with a CFX96 instrument (Bio-Rad, Hercules, CA, USA). Control samples representing all possible genotypes, along with a negative control, were included in each run.

### 2.4 Statistical analysis

Hardy-Weinberg equilibrium was tested for all the genetic polymorphisms distributions applying Chi-square or Fisher’s exact tests. Chi-squared statistics were applied to 2XN tables where appropriate to compare genotypic and allelic distributions of the SNPs between RR-MS + SP-MS and PP-MS courses as well as between MS patients and healthy controls (HC). P values were considered significant at <0.05, Bonferroni correction was applied for multiple comparisons when appropriate (p_c_). For *IL-1β rs1143634*, whose allelic and genotypic distributions differed in MS patients and in HC, statistical power calculations were performed using the minor allele frequency estimated from the healthy control population of the present study, assuming an allelic odds ratio (OR) of 1.5 and a two-sided α level of 0.05. ΔEDSS and ΔBI were calculated as multidisciplinary rehabilitation outcomes as follows: ΔEDSS= EDSS D- EDSS A, ΔBI= BI D-BI A. After Kolmogorov-Smirnov test calculated ΔEDSS and ΔBI resulted not normally distributed. One way ANOVA Kruskall-Wallis test or Wilcoxon-Mann-Whitney U test were applied when appropriate to test the association of SNPs’ genotypes with ΔEDSS and ΔBI scales. Linkage disequilibrium was calculated using the SHEsisPlus online software (http://shesisplus.bio-x.cn/SHEsis.html) (30). Associations between genetic polymorphisms and changes in disability scores (ΔEDSS, ΔBI) were also assessed using generalized linear models (GENLIN) with an identity link function and normal distribution. To account for the non-normality of residuals and obtain robust standard error estimates, the Huber–White sandwich estimator was applied. Covariates included sex, age, baseline EDSS or BI scores, disease duration and length of hospitalization. Genetic associations were primarily evaluated under an additive model by coding genotypes according to the number of minor alleles (0,1,2). Dominant models were subsequently explored when appropriate. *Post-hoc* power analyses were conducted for three genotype groups of each polymorphism using G*Power 3.1.9.7 (Heinrich-Heine-Universität Düsseldorf, Germany) (31, 32). The analyses were based on the appropriate statistical tests used in the study, assuming an alpha level of 0.05 and using effect sizes derived from the observed data. Cohen’s f was calculated for ANOVA to estimate the achieved statistical power, ensuring adequate sensitivity of the study to detect genotype-related differences in rehabilitation outcomes. The combined effect of *IL-1α rs3783521*, *IL-1β rs1143627* and *IL-1β 16944* was assessed with the online software SNPStats (https://www.snpstats.net/start.htm). All the statistical analyses were performed with SPSS software (version 29.0.1.0 (171), IBM, USA) and MedCalc (version 11.5.0.0, MedCalc software bvba).

## 3 Results

### 3.1 Study population

**Table 1** summarizes the demographic and clinical characteristics of the study population stratified by MS phenotype (RR-MS, PP-MS, and SP-MS). Significant differences were observed among groups in sex distribution, with a higher proportion of females in the RR-MS group compared with the PP-MS and SP-MS groups. PP-MS and SP-MS patients were significantly older than RR-MS patients, and age at disease onset was higher in PP-MS. Disease duration differed significantly among groups, being longer in SP-MS compared with RR-MS and PP-MS. No significant differences were observed in length of hospitalization across phenotypes. Both baseline and discharge EDSS scores showed a clear gradient of disability, with RR-MS patients exhibiting lower disability, and SP-MS patients showing the highest impairment, while PP-MS patients presented intermediate values. Similarly, mBI confirmed greater functional independence in RR-MS and reduced autonomy in progressive forms of MS. Overall, these findings confirm the expected clinical stratification across MS phenotypes (1).

**Table 1:** Study population demographics and clinical variables.

MS type	RR-MS	PP-MS	SP-MS	p value	Statistics
N (%)	43 (26.54)	30 (18.52)	89 (54.94)		
Males/Females, (N, %)	7/36 (16.28/83.72)	16/14 (53.33/46.67)	43/46 (48.31/51.69)	^a^ =0.001; ^b <^0.001; ^c^ 0.638	Chi-square test
Age, years (mean, SD)	46.91 (9.73)	56.50 (11.73)	55.02^b^ (12.62)	^a <^0.001; ^b <^0.001; ^c^ 0.574	One-way ANOVA
Age at onset, years (mean, SD)	26.44 (9.63)	38.21 (11.41)	29.32 (11.52)	^a <^0.001; ^b <^0.114; ^c <^0.001	One-way ANOVA
Disease duration, years (mean, SD)	20.47 (10.38)	17.59 (8.21)	25.59 (10.12)	^a^ =0.204; ^b =^0.008; ^c <^0.001	One-way ANOVA
Length of hospitalization, days (mean, SD)	37.49 (9.88)	33.83 (9.76)	36.74 (9.72)	^a^ =0.122; ^b^ =0.681; ^c^ 0.159	One-way ANOVA
EDSS A, (median, IQR)	6.00 (5.00-6.50)	6.50 (6.50-8.00)	7.00 (6.50-8.00)	^a <^0.001; ^b <^0.001; ^c^ 0.058	Kruskall Wallis test
EDSS D, (median, IQR)	5.50 (4.50-6.50)	6.50 (6.50-8.00)	6.50 (6.50-8.00)	^a <^0.001; ^b <^0.001; ^c^ 0.100	Kruskall Wallis test
BI A, (median, IQR)	75.00 (66.25-80.00)	61.50 (46.00-72.00)	55.00 (38.00-65.50)	^a^ 0.002; ^b <^0.001; ^c^ 0.045	Kruskall Wallis test
BI D, (median, IQR)	83.00 (75.00-88.50)	69.50 (59.00-81.00)	62.00 (42.75-75.00)	^a^ 0.002; ^b <^0.001; ^c^ 0.029	Kruskall Wallis test

RR-MS, relapsing–remitting multiple sclerosis; PP-MS, primary progressive multiple sclerosis; SP-MS, secondary progressive multiple sclerosis; SD, standard deviation; IQR, interquartile range; EDSS A, expanded disability status scale at admission; EDSS D, expanded disability status scale at discharge; BI A, Barthel index at admission; BI D, Barthel index at discharge.

^a^RR-MS vs PP-MS comparisons; ^b^RR-MS vs SP-MS comparisons; ^c^PP-MS vs SP-MS comparisons.

### 3.2 IL-1α rs3783521, IL-1β rs16944, rs1143627 and rs1143634 SNPs genotypic and allelic distribution

**Figure 1:**
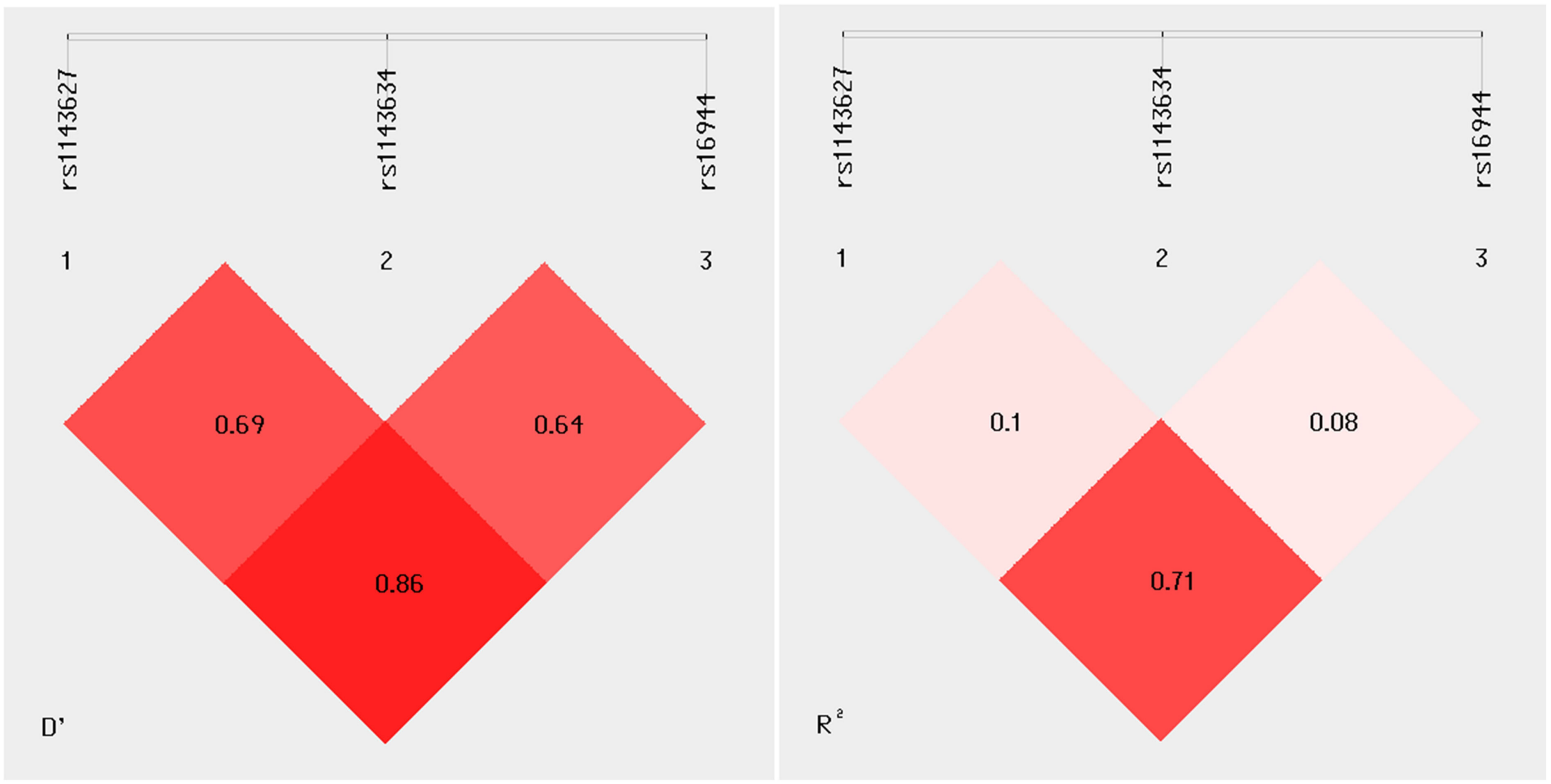
Linkage disequilibrium among IL-1β polymorphisms. Linkage disequilibrium plots showing pairwise relationships among IL-1β rs1143627, rs1143634, and rs16944 SNPs in the study population. D′ and r² values are reported within each block and indicate the strength of linkage disequilibrium between SNP pairs.

All the SNPs were in Hardy-Weinberg equilibrium: *IL-1α rs3783521* (p= 0.85), *IL-1β rs16944* (p= 1.00), *IL-1β rs1143627* (p= 0.86), *IL-1β rs1143634* (p= 0.71). *IL-1α rs3783521*, *IL-1β rs16944*, *rs1143627* and *rs1143634* SNPs genotypic and allelic distributions in MS patients are reported in **Table 2** (**Table 2**). From the genetic point of view, as SP-MS is the evolution of RR-MS, these patients were considered together (RR-MS + SP-MS) and were compared with PP-MS. No statistically significant differences were observed in genotypic and allelic distributions among MS phenotypes. Linkage disequilibrium (LD) analysis was calculated for *IL-1β rs16944*, *rs1143627* and *rs1143634* SNPs. Results are reported in **Figure 1**; LD between *rs16944*, *rs1143627* resulted moderate to strong (R’=0.71, D’=0.86) (**Figure 1**).

**Table 2:** Genotypic and allelic distributions of *IL-1α rs3783521*, *IL-1β rs1143627*, *rs1143634*, *rs16944* SNPs in the study population.

*IL-1α rs3783521*			
	RR-MS + SP-MS	PP-MS	p value
Genotype	N (%)	N (%)	
*AA*	14 (10.61)	1 (3.33)	
*AG*	52 (39.39)	14 (46.67)	
*GG*	66 (50.00)	15 (50.00)	
Total	132	30	0.424*
Allele			
*A*	80 (30.30)	16 (26.67)	
*G*	184 (69.70)	44 (73.33)	
Total	264	60	0.589
*IL-1β rs1143627*			
	RR-MS + SP-MS	PP-MS	p value
Genotype			
*AA*	59 (44.70)	14 (46.67)	
*AG*	58 (43.94)	13 (43.33)	
*GG*	15 (11.36)	3 (10.00)	
Total	132	30	0.969*
Allele			
*A*	176 (66.67)	41 (68.33)	
*G*	88 (33.33)	19 (31.67)	
Total	264	60	0.814
*IL-1β rs1143634*			
	RR-MS + SP-MS	PP-MS	p value
Genotype			
*AA*	15 (11.36)	1 (3.33)	
*AG*	51 (38.64)	15 (50.00)	
*GG*	66 (50.00)	14 (46.67)	
Total	132	30	0.297*
Allele			
*A*	81 (30.68)	17 (28.33)	
*G*	183 (69.32)	43 (71.67)	
Total	264	60	0.733
*IL-1β rs16944*			
Genotype	RR-MS + SP-MS	PP-MS	p value
*AA*	13 (9.85)	3 (10.0)	
*AG*	57 (43.18)	14 (46.67)	
*GG*	62 (46.97)	13 (43.33)	
Total	132	30	0.933*
Allele			
*A*	83 (31.44)	20 (33.33)	
*G*	181 (68.56)	40 (66.67)	
Total	264	60	0.771

*p_c_, p corrected for 2 degrees of freedom (Bonferroni’s correction); Statistics: Chi-square test for all comparisons.

RR-MS and SP-MS were considered as a unique group.

### 3.3 Comparison of SNPs genotypic and allelic distributions with Italian healthy controls published data

The genotypic and allelic distributions of *IL-1α rs3783521*, *IL-1β rs16944*, *rs1143627*, and *rs1143634* SNPs obtained in MS patients were compared with previously published data from Italian HC. Specifically, data for *IL-1α rs3783521*, were obtained from 439 HC (33); *IL-1β rs1143627* data were derived from 236 Italian blood donors (34); data for *IL-1β rs1143634* and *rs16944* were taken from a cohort of 663 Italian healthy athletes and non-athletes (35). Reference studies were selected based both on geographic origin and sample size criteria. Results are summarized in **Table 3** (**Table 3**). No significant differences were observed between MS patients and HC for *IL-1α rs3783521* or for *IL-1β rs16944* and *rs1143627*. In contrast, distribution of *IL-1β rs1143634* was significantly different in MS compared to HC (p_c_= 0.005). Thus, the *GG* genotype was more frequent in HC (61.54%) than in MS patients (49.38%) (p_c_= 0.01, OR = 0.61, 95%CI= 0.43-0.86) whereas the *AA* genotype was more prevalent in MS patients (9.88%) compared with HC (4.98%) (p_c_=0.05, OR = 2.09, 95%CI= 1.10-3.87). Consistently, allelic frequencies also differed significantly between the two groups (p= 0.001, OR = 1.56, 95%CI= 1.19-2.05). Under an allelic model, for *IL-1β rs1143634*, assuming a minor allele frequency of 0.22 and an odds ratio of 1.5, the study design had an estimated statistical power of approximately 80% at a significance level of 0.05.

**Table 3:** Genotypic and allelic distributions of *IL-1α rs3783521*, *IL-1β rs1143627*, *rs1143634*, *rs16944* SNPs in the study population and in published Italian HC datasets.

*IL-1α rs3783521*				
	MS	HC^§^	p value	OR (95%CI)
Genotype	N (%)	N (%)		
*AA*	15 (9.26)	38 (8.66)		
*AG*	66 (40.74)	203 (46.24)		
*GG*	81 (50.00)	198 (45.10)		
Total	162	439	0.482*	
Allele				
*A*	96 (29.63)	279 (31.78)		
*G*	228 (70.37)	599 (68.22)		
Total	324	878	0.478	
*IL-1β rs1143627*				
	MS	HC^§§^	p value	OR (95%CI)
Genotype	N (%)	N (%)		
*AA*	73 (45.06)	96 (40.68)		
*AG*	71 (43.83)	116 (49.15)		
*GG*	18 (11.11)	24 (10.17)		
Total	162	236	0.578*	
Allele				
*A*	217 (66.98)	308 (65.25)		
*G*	107 (33.02)	164 (34.75)		
Total	324	472	0.617	
*IL-1β rs1143634*				
	MS	HC^§§§^	p value	OR (95%CI)
Genotype	N (%)	N (%)		
*AA*	16 (9.88)	33 (4.98)	0.05*	2.09 (1.10-3.87)
*AG*	66 (40.74)	222 (36.82)	0.09	1.36 (0.96-1.94)
*GG*	80 (49.38)	408 (61.54)	0.01*	0.61 (0.43-0.86)
Total	162	663	0.005*	
Allele				
*A*	98 (30.25)	288 (21.72)		
*G*	226 (69.75)	1038 (77.97)		
Total	324	1326	0.001	1.56 (1.19-2.05)
*IL-1β rs16944*				
	MS	HC^§§§^		
Genotype	N (%)	N (%)	p value	OR (95%CI)
*AA*	16 (9.87)	68 (10.32)		
*AG*	71 (43.83)	308 (46.74)		
*GG*	75 (46.30)	283 (42.94)		
Total	162	659	0.741*	
Allele				
*A*	103 (31.79)	444 (33.69)		
*G*	221 (68.21)	874 (66.31)		
Total	324	1318	0.519	

*p_c_ corrected for 2 degrees of freedom (Bonferroni’s correction); ^§^Ferri C et al., 2000; ^§§^Fontanini E et al., 2010; ^§§§^Cauci S et al., 2010; Statistics: Chi-square test for all comparisons.

### 3.4 IL-1α rs3783521, IL-1β rs1143627, rs1143634 and rs16944 SNPs impact on EDSS and BI at baseline

EDSS and BI at baseline were not normally distributed after Kolmogorov-Smirnov test. Therefore, the Kruskal–Wallis test was used to assess whether the genotypes of the *IL-1α rs3783521*, *IL-1β rs1143627*, *rs1143634*, and *rs16944* SNPs influenced these parameters. At baseline, EDSS was not significantly influenced by IL-1α rs3783521 (p = 0.298), IL-1β rs1143627 (p = 0.655), or rs16944 (p = 0.750). In contrast, IL-1β rs1143634 was associated with EDSS (p = 0.035). When patients were stratified into RR-MS and progressive MS (SP-MS + PP-MS) groups, no significant associations were observed between EDSS and any of the SNPs (data not shown).

The same analyses were performed for BI. At baseline, BI was not significantly influenced by IL-1α rs3783521 (p = 0.146), IL-1β rs1143627 (p = 0.718), or rs16944 (p = 0.472). However, IL-1β rs1143634 showed a significant association with BI (p = 0.017). After stratification of patients as described above, no significant associations were observed between BI and any of the SNPs (data not shown).

### 3.5 *IL-1α rs3783521*, *IL-1β rs1143627*, *rs1143634* and *rs16944* SNPs impact on multidisciplinary rehabilitation outcomes

ΔEDSS= EDSS D-EDSS A, ΔBI= BI D-BI A were calculated as multidisciplinary rehabilitation outcomes; these calculated variables resulted to be not normally distributed after Kolmogorov-Smirnov test. One way ANOVA Kruskall-Wallis test or Wilcoxon-Mann-Whitney U test were applied when appropriate as explorative analyses to test the association of SNPs’ genotypes under additive and dominant models with ΔEDSS and ΔBI scales. Results are reported in **Table 4** (**Table 4**). ΔEDSS resulted to be influenced by *IL-1α rs3783521* (p_c_= 0.012), *IL-1β rs1143627* (p_c_= 0.002), *IL-1β rs1143634* (p_c_=0.031) and *IL-1β 16944* (p_c_= 0.002). In detail, for *IL-1α rs3783521* there was a difference of ΔEDSS between *GG* (average rank= 89.50) and *AG* genotypes’ carriers (average rank= 72.50) (p_c_= 0.010) and considering the dominant model (*GG* vs. *AG*+*AA*) the difference in ΔEDSS between *GG* and *AG*+*AA* carriers (average rank= 73.42) augmented (p=0.003). For *IL-1β rs1143627* there was a significative difference in ΔEDSS between *AA* (average rank= 71.25) and *AG* carriers (average rank=91.86) (p_c_= 0.001). Considering the dominant model (*AA* vs *AG*+*GG*), also in this case, the difference in ΔEDSS between *AA* and *AG*+*GG* (average rank= 89.91) carriers augmented (p<0.001). For *IL-1β rs1143634*, under additive model after Bonferroni’s correction, the difference in ΔEDSS between *AA* and *GG* was not statistically significant (p_c_= 0.062), but under the dominant model (*GG* vs *AG*+*AA*), the difference in ΔEDSS between *GG* (average rank= 75.10) and *AG*+*AA* carriers (average rank= 88.06) remained significant (p= 0.019). Finally, for *IL-1β 16944* there was a difference in ΔEDSS between *GG* (average rank= 71.01) and *AG* carriers (average rank= 90.54) (p_c_= 0.002) and when comparing with the dominant model *GG* vs. *AG*+*GG* carriers (average rank= 90.55), the difference augmented (p<0-001). For what concern ΔBI, only *IL-1β rs1143634* showed to have an influence. Under dominant level, *AA*+*AG* carriers had a higher ΔBI compared to *GG* carriers (p= 0.013).

**Table 4:** ΔEDSS and ΔBI alongside *IL-1α rs3783521*, *IL-1β rs1143627*, *rs1143634*, *rs16944* SNPs genotypes in the study population.

*IL-1α rs3783521*					
Genotype	N	ΔEDSS average rank	p value	ΔBI average rank	P value
Additive model					
*AA*	15	77.47		73.33	
*AG*	66	72.50	0.010*	87.36	
*GG*	81	89.50	0.010*	78.23	
Total	162		0.012*		0.384
Dominant model					
*AA*+*AG*	81	73.42		84.77	
*GG*	81	89.58		78.23	
Total	162		**0.003**		0.371
*IL-1β rs1143627*					
Genotype	N	ΔEDSS average rank	P value	ΔBI average rank	P value
Additive model					
*AA*	73	71.25	0.001*	87.95	
*AG*	71	91.86	0.001*	75.56	
*GG*	18	82.22		78.78	
Total	162		0.002*		0.269
Dominant model					
*AA*	73	71.25		87.95	
*AG*+*GG*	89	89.91		76.21	
Total	162		**<0.001**		0.110
*IL-1β rs1143634*					
Genotype	N	ΔEDSS average rank	P value	ΔBI average rank	P value
Additive model					
*AA*	16	65.84	0.062*	99.91	0.091*
*AG*	66	77.35		88.17	
*GG*	80	88.06	0.062*	72.32	0.091*
Total	162		0.031*		0.030*
Dominant model					
*AA*+*AG*	82	88.06		90.46	
*GG*	80	75.10		72.32	
Total	162		**0.019**		**0.013**
*IL-1β rs16944*					
Genotype	N	ΔEDSS average rank	P value	ΔBI average rank	P value
Additive model					
*AA*	16	90.56		69.03	
*AG*	71	90.54	0.002*	75.85	
*GG*	75	71.01	0.002*	89.51	
Total	162		0.002*		0.110*
Dominant model					
*AA*+*AG*	87	90.55		74.60	
*GG*	75	71.01		89.51	
Total	162		**<0.001**		0.042

p_c_ corrected for 2 degrees of freedom (Bonferroni’s correction); Statistics: Kruskall Wallis test for the comparisons of three groups, Mann–Whitney U test for the comparisons of two groups.

Bold values are the statistically significant p values.

Generalized linear models with identity link and robust (Huber–White sandwich) variance estimators were then applied to confirm and extend the results of the non-parametric analyses, allowing adjustment for potential confounding variables (such as sex, age, baseline EDSS, baseline BI, disease duration, and hospitalization length). Genetic associations were primarily evaluated under an additive model, with dominant models explored when appropriate. IL-1α rs3783521 showed a consistent association with rehabilitation outcome, with the A allele being associated with greater improvement in ΔEDSS under both additive (B= −0.102, 95% CI −0.176 to −0.028, p = 0.007). Under the dominant model, carriers of at least one *A* allele (*AA*+*AG*) also showed greater improvement compared with non-carriers (*GG*) (B = −0.156, 95% CI −0.245 to −0.067, p < 0.001). In the same model, longer hospitalization was independently associated with greater improvement (B= −0.006, 95% CI −0.011 to −0.001, p = 0.028), independently of disease duration, sex, age and baseline EDSS.

For IL-1β rs1143627, no significant association was observed under the additive model after adjustment; however, under the dominant model, carriers of at least one *G* allele (*AG*+*GG*) showed significantly less improvement compared to *AA* individuals (B = 0.143, 95% CI 0.053 to 0.233, p = 0.002), suggesting a detrimental effect of the *G* allele, independently of age, baseline EDSS, disease duration, and hospitalization length. Female gender resulted in augmented improvement (B= -0.100, 95% CI -0.186 to -0.013, p=0.024).

*IL-1β rs1143634* was associated with ΔEDSS under the additive model, with the *A* allele correlating with greater improvement (B= −0.070, 95% CI −0.135 to −0.005, p = 0.036). Consistently, under the dominant model, carriers of at least one *A* allele (*AA*+*AG*) showed greater improvement compared to *GG* individuals (B= −0.097, 95% CI -0.184 to -0.010, p = 0.028). In this model, female sex showed greater improvement (B= −0.086, 95% CI -0.170 to -0.001, p = 0.048), age baseline EDSS, disease duration and length of hospitalization resulted to not influence improvement.

Finally, IL-1β rs16944 showed a robust association with rehabilitation outcome, with the *A* allele being consistently associated with reduced improvement in ΔEDSS under additive model (B = 0.094, 95% CI 0.032 to 0.157, p = 0.003). Under dominant model, carriers of at least one *A* allele (*AA*+*AG*) showed the worst outcome (B = 0.137, 95% CI 0.050 to 0.223, p = 0.002), while the G allele appears to be associated with a more favorable rehabilitation outcome. In addition, female sex was independently associated with greater improvement (B = −0.089, 95% CI −0.177 to −0.002, p = 0.045). Age, disease duration length of hospitalization and baseline EDSS did not influence improvement in this model.

The same analyses were performed for ΔBI, but no significant associations were found between the genetic polymorphisms and this parameter.

To assess the robustness of these findings, *post-hoc* power analyses were performed using one-way ANOVA to compare ΔEDSS across the three genotype groups for each SNP. For *IL-1α rs3783521*, the analysis yielded a moderate effect size (Cohen’s f= 0.275) and high statistical power (approximately 93%) at an alpha level of 0.05. Similarly, IL-1β rs1143627 showed a comparable effect size (f= 0.271), corresponding to a *post-hoc* power of 91%. The IL-1β rs16944 polymorphism also demonstrated a moderate effect size (f= 0.270) with adequate power (83%). These findings indicate that the study was sufficiently powered to detect genotype-related differences in rehabilitation outcomes for *IL-1α rs3783521* and *IL-1β rs1143627* and *rs16944* polymorphisms. In contrast, *IL-1β rs1143634* exhibited a small-to-moderate effect size (f= 0.152) and limited statistical power (56% at α= 0.05), suggesting reduced sensitivity to detect differences across all three genotype groups for this variant.

Additional stratified analyses dividing patients by clinical phenotype into RR-MS and progressive MS (SP-MS + PP-MS) were considered but *post-hoc* power calculations indicated insufficient statistical power in the RR-MS subgroup due to the limited sample size for most of the SNPs (data not shown). Therefore, to avoid unreliable results, analyses were conducted only on the overall cohort.

**Figure 2:**
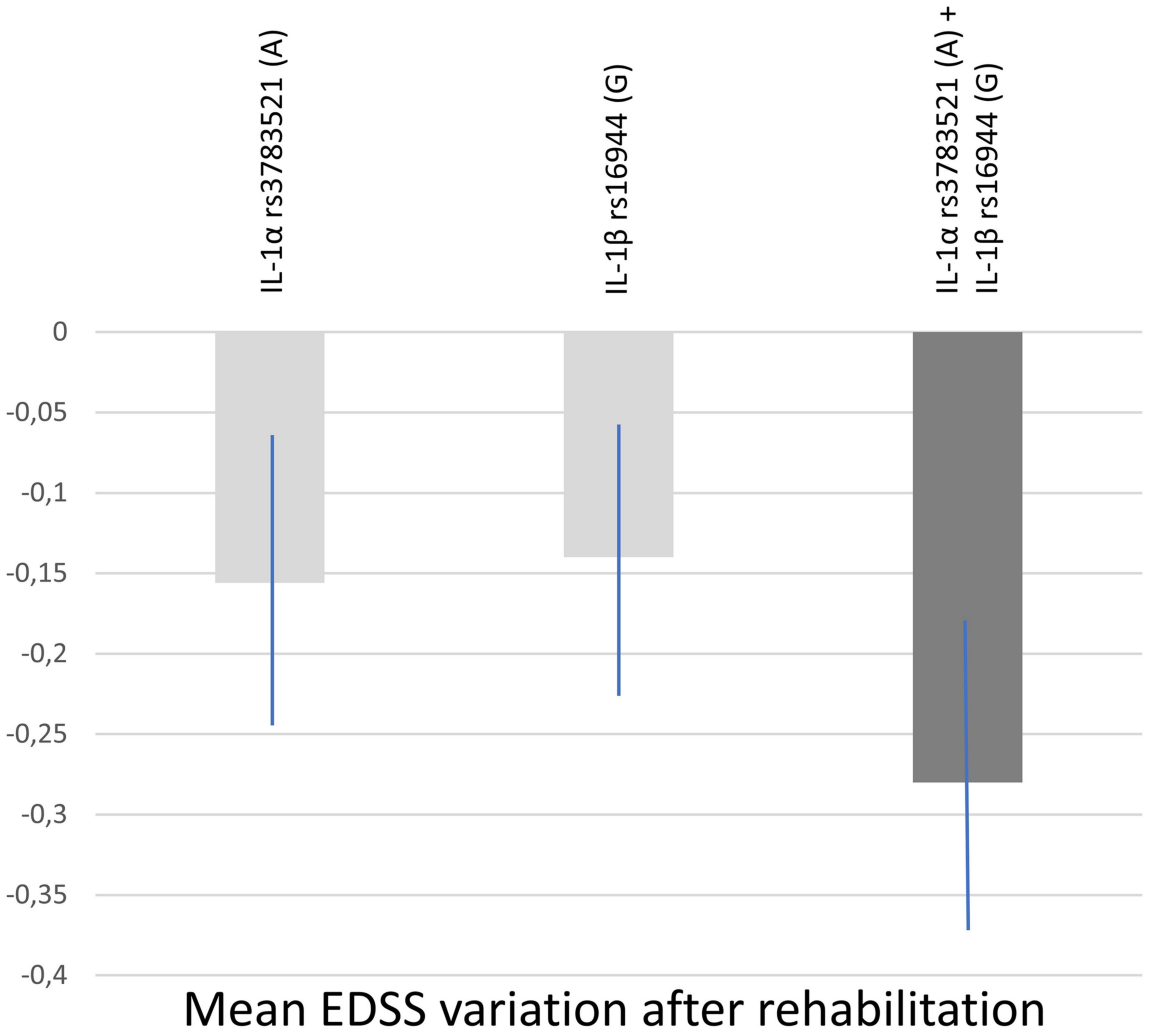
Mean EDSS improvement after multidisciplinary rehabilitation according to IL-1 genotype. Mean ΔEDSS values (EDSS at discharge − EDSS at admission) in carriers of the IL-1α rs3783521 A allele, the IL-1β rs16944 G allele, and both alleles. More negative ΔEDSS values indicate greater improvement in disability. Error bars represent standard errors of the mean. Carriers of both favorable alleles showed the largest improvement following multidisciplinary rehabilitation.

In summary, based on these robust statistical analyses and on the statistical power of the sample size for each SNP, the alleles more strongly and with a higher likelihood associated with improved rehabilitation outcomes were *IL-1α rs3783521 A* and *IL-1β 16944 G*. *IL-1β rs1143627* resulted to be associated with multidisciplinary rehabilitation outcome only under dominant model, not under additive model. The combined effect of these variants and its influence on ΔEDSS was then evaluated. Individuals carrying the combination of “favorable” alleles *IL-1α rs3783521 A* and *IL-1β 16944 G* showed the greatest improvement in disability. This allele combination, with a frequency of 0.148 in the study population, was associated with a mean ΔEDSS of −0.280 (95% CI: −0.370 to −0.180; p < 0.0001). In **Figure 2** a graphical representation of the effect of each 2 SNP alleles and the combination of the 2 on ΔEDSS is presented (**Figure 2**).

## 4 Discussion

MS is a major cause of neurological disability in young adults and frequently results in progressive functional impairment requiring multidisciplinary rehabilitative care. In this context, the identification of biological factors potentially associated with rehabilitation outcomes may be clinically relevant. In this pilot study we investigated whether selected *IL-1α* and *IL-1β* genes SNPs associate with both susceptibility to MS and rehabilitation outcome in a cohort of Italian patients undergoing inpatient multidisciplinary rehabilitation; none of whom were receiving disease modifying treatments during the period of rehabilitation that might have influenced the results. To evaluate the role of *IL-1α* and *IL-1β* genes SNPs in MS susceptibility, we compared the genotypic and allelic distributions of the studied polymorphisms in our overall MS cohort with those reported in the literature. The *IL-1β rs1143634 GG* genotype and *G* allele were identified as protective factors against MS. Consistently, a previous study in an Iranian population reported the *IL-1β rs1143634 AA* genotype and *A* allele as risk factors for MS (36). In contrast, other studies, including results of a meta-analysis found no significant association between *rs1143634* and MS (37). These discrepancies may be explained by ethnic differences between populations and will need to be analyzed in future studies. Comparisons between RR-MS and PP-MS subtypes revealed no significant differences in the distributions of these polymorphisms. Baseline EDSS and BI clinical outcomes were analyzed according to SNP genotypes to explore potential associations with *IL-1α* and *IL-1β* polymorphisms. Although *IL-1β rs1143634* showed a nominal association with EDSS A and BI A, this finding should be interpreted with caution due to the limited statistical power of the *post hoc* analyses for this SNP.

The possible association of the same *IL-1α* and *IL-1β* SNPs with improvements in ΔEDSS (EDSS D- EDSS A) and ΔBI (BI D- BI A) following multidisciplinary rehabilitation was analyzed next. Results showed that polymorphisms in *IL-1α* and in *IL-1β* associate with rehabilitation outcome as analyzed by ΔEDSS. In particular, the *IL-1α rs3783521 A* and *IL-1β rs16944 G* alleles associate with a significantly better rehabilitation outcome.

Regarding the functional impact of the associated SNPs on gene expression, eQTL analysis from the GTEx database indicates that the *IL-1α rs3783521 AA* genotype is associated with reduced *IL-1α* expression across multiple tissues, including skin (both sun-exposed and non–sun-exposed), spleen, testis, esophageal mucosa, and pituitary gland (GTEx Portal, 2026. https://gtexportal.org/home/) (38). This could justify our findings: the *A* allele which is associated with better rehabilitation outcome results in reduced *IL-1α* expression and consequent down-modulation of inflammation. The *IL-1β* promoter variant *rs1143627* instead converts a non-canonical TBP-binding site into a canonical TATA box with carriers of the minor *A* allele showing significantly higher TBP affinity and consequently increased *IL-1β* expression compared with the G allele (39). Notably, *IL-1β* expression was shown to be significantly increased in individuals with the *rs16944 AA* genotype whereas it was reduced in *GG* carriers (38). The combined effect of *rs1143627* and *rs16944* alleles and genotypes on *IL-1β* gene functionality will nevertheless need to be explored further.

IL-1 cytokines are known to be key mediators of neuroinflammation in MS, influencing both demyelination and axonal damage (40). While IL-1α is constitutively expressed in many cell types and can amplify inflammatory responses, IL-1β is an inducible cytokine, primarily produced by blood myeloid cells, pathogenic lymphocytes, and CNS-resident microglia and astrocytes during autoimmune and neurodegenerative processes (41). It should be emphasized that IL-1β has pleiotropic effects and plays important roles in normal brain function: despite its potent pro-inflammatory function. Thus, despite its potent pro-inflammatory functions, IL-1β can also contribute to neuroprotection, tissue remodeling, and repair (42). Accordingly, whether IL-1β initiates damage or contributes to repair likely depends on the local context, including its concentration and the surrounding environmental milieu (43, 44). These context-dependent mechanisms may plausibly modulate the CNS capacity to respond to rehabilitative interventions aimed at promoting functional recovery andgenetic polymorphisms associated with lower IL-1 expression may facilitate rehabilitation-induced neuroplastic changes. Comparison with existing literature is limited, as we did not find other similar studies. However, our findings are in line with previous observations in other neurological conditions. In patients with traumatic brain injury, carriers of the IL-1B rs16944 A allele were more frequently represented among individuals with poor functional outcomes (45), suggesting that IL-1 genetic variability may influence recovery processes across different forms of neurological injury.The present study adds novel evidence that genetic differences influence non-pharmacological recovery processes, reinforcing the concept of personalized rehabilitation strategies tailored to individual genetic profiles. The inclusion of patients not receiving disease-modifying therapies allowed us to minimize pharmacological confounding factors, resulting in a more accurate analysis of the contribution of IL-1 genetic variability to rehabilitation outcomes. Several limitations should nevertheless be acknowledged. The selection of a limited number of *IL-1α* and *IL-1β* genes SNPs for analysis was guided by prior literature. However, a more comprehensive investigation should include a broader range of polymorphisms, as the combined effects of multiple variants is likely to shed further light on the functional impact on cytokine expression on disease outcomes. Moreover, our study is monocentric and lacks an independent validation cohort, which may limit generalizability. We plan to validate these results in larger cohorts with sufficient sample size to ensure adequate statistical power even after stratification by MS clinical phenotype. Results will allow a more reliable assessment of phenotype-specific genetic effects on rehabilitation outcomes. We will also assess *IL-1α* and *IL-1β* gene expression and concentration in biologic fluids before and after treatment to further elucidate their role in disease mechanisms and rehabilitation response.

In conclusion, our findings suggest that *IL-1α* and *IL-1β* genetic variability associates with functional recovery following multidisciplinary rehabilitation in MS. While these results provide preliminary insight into the potential influence of genetic background on rehabilitation outcomes, further studies are needed to confirm these observations. The integration of genotyping into personalized rehabilitation planning may represent a possible future direction, pending additional evidence.

## Data Availability

The datasets presented in this study can be found in online repositories. The names of the repository/repositories and accession number(s) can be found below: https://zenodo.org/records/19817695, https://zenodo.org/records/19817695.
